# EWS–FLI1‐targeting peptide identifies Ewing sarcoma tumor boundaries and lymph node metastasis via near‐infrared imaging

**DOI:** 10.1002/1878-0261.13081

**Published:** 2021-08-30

**Authors:** Yu Wang, Hengtang Mai, Ying Yuan, Hairen Chen, Song Wu, Xiang Hu, Aixi Yu

**Affiliations:** ^1^ Department of Orthopaedic Trauma and Microsurgery Wuhan University Zhongnan Hospital China; ^2^ Hubei Province Engineering and Technology Research Center for Fluorinated Pharmaceuticals School of Pharmaceutical Sciences Wuhan University China

**Keywords:** Ewing sarcoma, EWS–FLI1, near‐infrared imaging, surgical guidance, targeting peptide

## Abstract

Ewing sarcoma (ES) is one of the most aggressive types of pediatric tumors. The lack of tools for the identification of ES has largely hindered clinical diagnosis and the improvement of treatment. To address this challenge, we synthesized a near‐infrared (NIR) fluorescent probe (CS2‐N‐E9R) that targets the ES‐specific fusion protein EWS–FLI1 (E/F). This probe exhibited specific and high binding affinity to E/F. Further studies in animal models showed that CS2‐N‐E9R can be used to identify the boundaries of ES and lymph node metastases under a complex biological environment. These results demonstrate that CS2‐N‐E9R is a promising probe for early diagnosis and surgical guidance of ES through molecularly targeted NIR imaging.

## Graphical abstract

To address the clinical dilemma in the diagnosis of recurrent and metastatic Ewing sarcoma (ES), the authors synthesized the first near‐infrared fluorescent probe that targets the ES‐specific fusion protein EWS–FLI1 (CS2‐N‐E9R). CS2‐N‐E9R provides the potential to realize noninvasive diagnosis of orthotopic ES and lymph node metastases and can also identify ES tumor boundaries to guide accurate surgical resection.

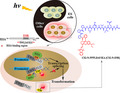

**Abbreviations**
Avg.: average
CCK‐8: Cell Counting Kit‐8
CIMs: conventional imaging modalities
CON: control
DMF: dimethylformamide
DTT: dl‐dithiothreitol
E/F: EWS–FLI1
ES: Ewing sarcoma
FL: fluorescence
Fmoc: fluorenylmethoxycarbonyl
HBTU: 2‐(1H‐Benzotriazole‐1‐yl)‐1,1,3,3‐tetramethyluronium hexafluorophosphate
HRMS: high‐resolution mass spectra
LMN: lymph node metastasis
NIR: near‐infrared
op: operation
RHA: RNA helicase A
ROI: region‐of‐interest
TFA: trifluoroacetic acid

## 1 Introduction

Ewing sarcoma (ES) is a major form of pediatric tumor that poses large threat to adolescent health, with 80% of ES patients are under the age of 20 [1, 2, 3]. Advancements in surgery, systemic multi‐agent chemotherapy, and irradiation have increased survival to 68% at 5 years over the past three decades [4]. However, little progress has been made for ES patients with metastasis or recurrence; this subset has a poor 5‐year survival rate below 25% [5, 6, 7]. One of the major clinical challenges is the lack of effective tools to help surgeons accurately characterize metastatic and recurrent lesions in the early stage, thus hindering the accurate diagnosis and reasonable treatment of this fatal disease [8].

Ewing sarcoma can frequently masquerade as other bone and soft tissue sarcoma (rhabdomyosarcoma and osteosarcoma) [9], bone infection (osteomyelitis) [10, 11] or other cancers involved bones (lymphoma) [12, 13]. At present, the diagnosis and differential diagnosis of ES are based on tissue biopsy [14]. Noninvasive examinations, such as conventional imaging modalities (CIMs) including radiography [15, 16], contrast‐enhanced MRI [17, 18] and contrast‐enhanced thoracic helical CT [19, 20] are lack of specificity and cannot accurately distinguish ES from other bone and soft tissue sarcomas [21]. Meanwhile, for the treatment of localized ES, surgical removal of the tumor and sentinel lymph node if possible [22]. The goal of the operation is to perform a thorough resection of tumor tissue along with additional cuff of normal tissue for safe measure [23], and many subjects face amputation or endo‐prosthetic surgery; these procedures have enormous psychological and financial repercussions [24, 25]. Consequently, there is a great demand for the development of noninvasive tools for ES diagnosis and accurate resection guidance with high spatiotemporal resolutions and high sensitivity.

Near‐infrared (NIR) fluorescence imaging technology has gained considerable attraction in biomedical sciences in recent years [26]. It is well known that NIR emission from 650 to 900 nm has the biological merits of less photodamage, deep tissue penetration, and less auto‐fluorescence interference created by biomolecules in the living system [27]. At present, NIR probes are promising in the field of noninvasive tumor imaging [28, 29]. NIR imaging can compensate for the inability of CIMs to accurately diagnose sarcoma subtypes, and the inability to detect multiple tiny metastatic lesions, which is particularly important for subsequent tumor genotyping and the development of surgery and chemotherapy regimens [30]. In addition, NIR imaging can also achieve accurate surgical resection in real‐time fluorescent intervention during surgery, which is a promising auxiliary means to achieve complete resection of malignant sarcomas.

**Fig. 1:**
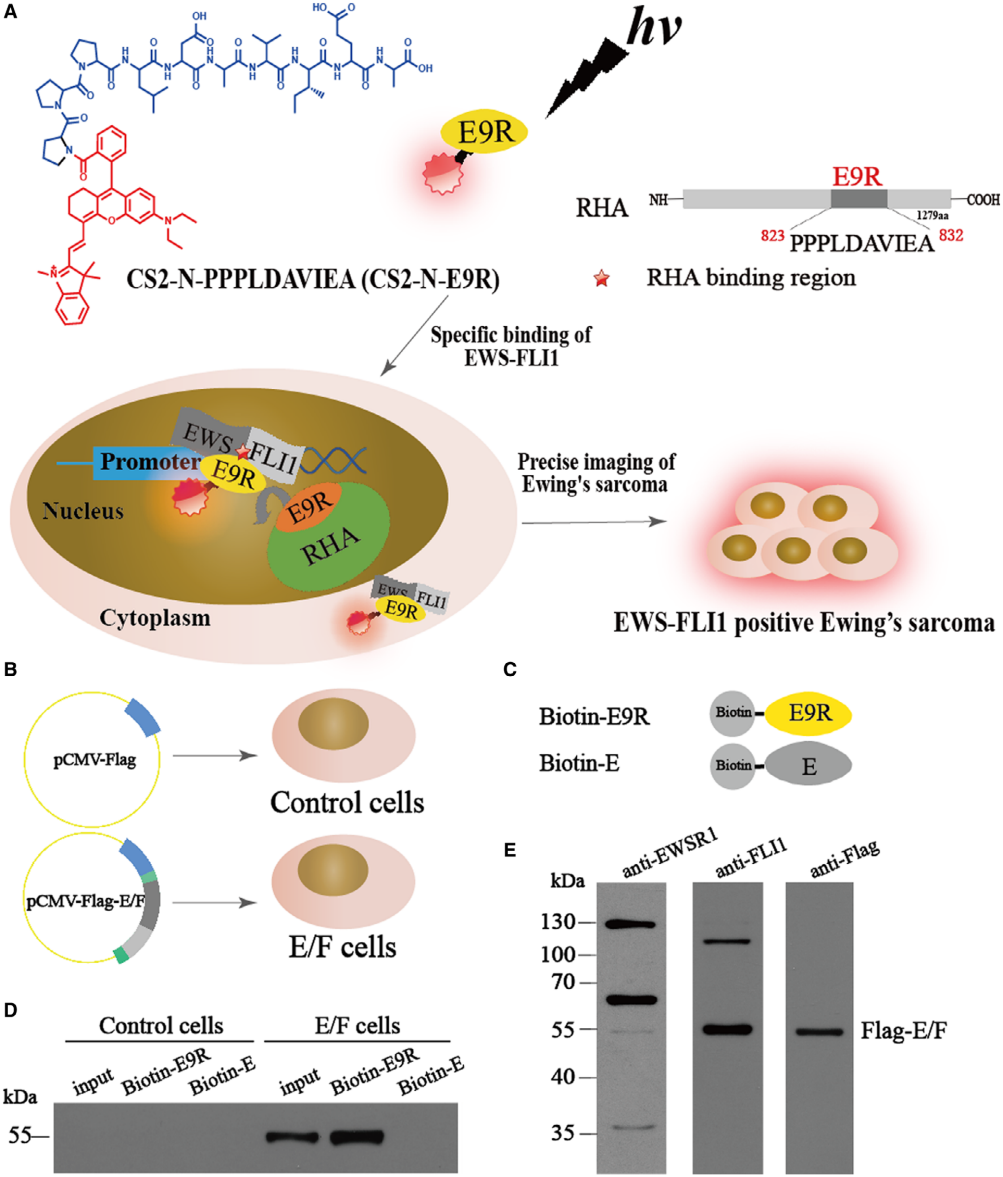
(A) Illustration of the competitive binding to E/F between RHA and CS2‐N‐E9R, and targeted imaging of ES cells with CS2‐N‐E9R. (B) Construction of the stable E/F‐expressing cells. (C) Schematic representation of pull‐down reagents used in the assay. (D) Western blot analysis of Flag‐E/F in the input and Flag‐E/F pull‐down products (*n* = 3 for each cell). (E) Anti‐EWSR1, anti‐FLI1 and anti‐Flag antibodies were used to verify the expression of Flag‐E/F in biotin‐E9R pull‐down products (*n* = 3 for each cell).

Ewing sarcoma has a characteristic t(11; 22)(q24; q12) chromosomal translocation that translates into the E/F oncogenic fusion protein [31]. Clinical trials show that this particular protein occurs in 90% of tumor specimens from ES patients [32]. E/F is a transcription factor that constitutes a transcriptional complex along with RNA helicase A (RHA) in nucleus and further affects the proliferation of ES by regulating DNA transcription and RNA splicing [33]. RHA binds to E/F at a specific region RHA_823–832_ with the sequence of PPPLDAVIEA (E9R) [34], which may benefit to construct a NIR probe for E/F imaging through tethering E9R and a NIR fluorophore. We assumed such a probe may competitively bind to E/F with RHA and thus light up the nucleus of ES cells. Furthermore, since E/F is also highly expressed in the cytoplasm of ES cells, this probe is considered to exert its functions in the cytoplasm as well. Given the above dual binding effects, we believe this probe may largely enhance the sensitivity and specificity for ES detection, which will eventually improve the diagnosis of this tumor. Based on the above assumptions, we introduced a NIR cyanine dye CS‐2 [35] into this peptide to form a NIR imaging probe (CS2‐N‐E9R), which can achieve precisely targeted imaging of E/F‐positive ES, accurately definition of tumor boundaries and detection of lymph node metastases (see Fig. 1A).

## 2 Materials and methods

### 2.1 General information and materials

All reagents used in this article were obtained from commercial suppliers and were used without further purification unless specially indicated. Solvents used were purified via standard methods. Twice‐distilled purified water used in all experiments was from Milli‐Q systems (Millipore, Billerica, MA, USA). NIR fluorophore CS‐2 was prepared according to the previous literature [35]. Peptide E9R (PPPLDAVIEA) and E (QSSSYGQQND) were prepared by Bioyeargene (Wuhan, China). High‐performance liquid chromatography was carried out on Thermo Scientific UltiMate HPLC system. Dynabeads™ M‐280 Streptavidin (Thermo Fisher Scientific, Waltham, MA, USA) was used in the pull‐down assay. ^1^H NMR spectra were collected by using 400 MHz Bruker spectrometer. Mass spectra were recorded on a Bruker TOF‐MS for high‐resolution mass spectra (HRMS). Absorption and fluorescence spectra were conducted by using UV‐Vis spectrophotometer (Shimadzu UV‐2600, Shimadzu, Kyoto, Japan) and fluorometer (Hitachi F‐4600, Hitachi, Tokyo, Japan), respectively. Instruments used in cell imaging tests were carried out on Leica‐LCS‐SP8‐STED confocal microscope. Pathological examinations were determined by Olympus IX73‐inverted microscope. Fluorescence intensity of microplates was detected by Odyssey® Infrared Imager (LI‐COR Biosciences, Lincoln, NE, USA). Cytotoxicity of probe was tested on a microplate reader (Thermo Fisher Scientific). *In vivo* imaging was performed on Bruker Xtreme BI.

### 2.2 Synthesis of CS2‐N‐E9R and CS2‐N‐E

The peptide E9R was synthesized according to the standard fluorenylmethoxycarbonyl (Fmoc) solid‐phase synthesis protocol using rink amide resin (Novabiochem, San Diego, CA, USA). All amino acids (10 equiv) were attached to the resin by stepwise elongation with 1‐hydroxybenzotriazole (10 equiv), 2‐(1H‐Benzotriazole‐1‐yl)‐1,1,3,3‐tetramethyluronium hexafluorophosphate (10 equiv), and *N*,*N*‐diisopropylethylamine (20 equiv) as coupling reagents in dimethylformamide (DMF, 15 mL). The Fmoc protecting groups were removed by 20% (V/V) of piperidine in DMF (15 mL). After the last amino acid in sequence was attached, the Fmoc protecting group was removed and the peptide was cleaved from the resin with 2 mL trifluoroacetic acid for 2 h. The solution was then evaporated under reduced pressure and purified by HPLC. The final product was characterized by HRMS. The synthetic procedure of CS2‐peptides is identical to the synthesis of the peptide until the last amino acid in sequence was attached and the protecting group Fmoc removed. The terminal free amino group was then reacted with the dye CS‐2 in 2 mL DMF/4 drops of *N*,*N*‐diisopropylethylamine for 2 h. The solution was evaporated and purified by HPLC. CS2‐peptides were characterized by HRMS. For the detailed information, see Figs S1–S8.

### 2.3 Optical measurements of probes

All the absorbance and fluorescence spectra were obtained at room temperature with increment set to 1 nm. Probe at 10 µM concentration was prepared for all optical measurements. Fluorescence spectra were collected at the excitation wavelengths of 690 nm slit widths, which were set to 10 nm. All solutions were prepared freshly for each measurement. Citrate–phosphate buffer was used for preparation from pH 3 to pH 8, while carbonate–bicarbonate buffer was made for pH 9 and pH 10.

### 2.4 Cells culture and cytotoxicity assays

Ewing sarcoma cells (A673 cells, RD‐ES cells), osteosarcoma cells (143B cells), hepatoma carcinoma cells (Hep3B cells), and primary embryonic human kidney cells (293T cells) were purchased from American Type Culture Collection (ATCC, Manassas, VA, USA). Cells treated with Dulbecco's modified Eagle's medium (Invitrogen, Carlsbad, CA, USA), supplemented with 10% FBS (Invitrogen). Cells were seeded in culture dishes and then incubated at 37 °C under a humidified atmosphere containing 5% CO_2_ and were passaged upon reaching 90% confluence. Cell Counting Kit‐8 (CCK‐8) was used to test the measurement of cell viability. A673, RD‐ES, 143B, Hep3B, and 293T cells were seeded in 96‐well microplates, respectively, at a density of 3 × 10^4^ cells·mL^−1^ with 150‐μL medium containing 10% FBS. After 24 h of cell attachment, the plates were then washed three times with 100 μL per well PBS. The cells were then cultured in medium with 0, 1, 5, 10, 15, and 20 μm of probe CS2‐N‐E9R for 24 h. Six replicate wells were used for each control and test concentration. 10 μL of CCK‐8 solution was added to each well, and the plates were incubated at 37 °C for another 1 h in a 5% CO_2_ humidified incubator. Optical density of solutions was determined on microplate reader at 450 nm.

### 2.5 Construction of stable expression colons

*Ews‐fli1* was subcloned into pCMV‐3Tag‐1A vector by BamHⅠ/XhoⅠ restriction sites to construct eukaryotic expression vector pCMV‐Flag‐E/F. This vector also encodes 3×Flag epitope for the detection of Flag‐tagged fusion protein. The constructed vector was verified by agarose gel electrophoresis to confirm the size of the cloned fragment, and gene sequencing confirmed the flanking sequences of the cloning site was correct (Fig. S9). After that transfect, the successfully constructed pCMV‐Flag‐E/F into 293T cells by lipofection method to obtain stably expressing E/F cells and also transfect the empty pCMV‐3Tag‐1A vector (pCMV‐Flag) into 293T cells as control cells. Western blot was used to verify the expression of Flag‐E/F in E/F cells.

### 2.6 Pull‐down assay

For the pull‐down assay, cells were lysed using 500 μL RIPA Lysis Buffer containing 25 mm HEPES and 5 μL protease inhibitor cocktail. The lysed cell sample was centrifuged at 12,000 g for 10 min at 4 °C, and the supernatant was added with 10 μL biotinylated peptides biotin‐E9R or biotin‐E (as a control) and incubated for 2 h at room temperature. Later, 50 μL Streptavidin beads were added and incubated at 4 °C overnight. The Streptavidin beads mixture was washed twice with 500 μL elution buffer and discard the supernatant and added 10 μL SDS‐loading dye containing 10 mm dl‐dithiothreitol to prepare samples for SDS/PAGE. The samples were then loaded onto a 10% SDS/PAGE and transferred to nitrocellulose membrane. Antibodies against Flag were used to detect the pull‐down of Flag‐tagged E/F fusion protein.

### 2.7 Colocalization imaging

A673 cells, RD‐ES cells, and 143B cells were seeded in confocal culture dishes at approximately concentration of 2 × 10^5^ cells with 1.5‐mL medium and allowed to culture for 24 h and then added 200 μL of 4% formaldehyde fixing solution to dishes and sit for 20 min. We remove fixing solution and permeabilize the cells by adding 200 μL of Triton X‐100 permeabilization solution to each dish for 10 min, then wash three times with 1 mL PBS. We blocked the dishes by adding 150 μL of Odyssey Blocking Buffer and incubated for 1 h. Subsequently, 50 μL of primary antibodies EWSR1 or FLI1 (1 : 300 dilution) was added into the confocal dish at 4 °C gently shaking overnight. After washed three times, 50 μL of secondary antibody conjugated to FITC (1 : 500 dilution) and 10 μm of probe CS2‐N‐E9R were added to dishes. All dishes were protected from the light, and following by 1 h gently shaking, dishes were washed three times by 1 mL PBS. After that, two drops of DAPI dye were added into confocal dishes for the next phase of confocal fluorescence imaging.

### 2.8 Probes binding affinity, time‐dependent, and dose‐dependent living cell imaging

As to the binding affinity test, serial concentrations of E9R (from 4 × 10^−3^ m to 4 × 10^−9^ m) were prepared as competitors for CS2‐N‐E9R. A673 cells were seeded into 96‐well Black Polystyrene Assay plate at a concentration of 4 × 10^3^ cells per well with 150 μL medium containing 10% FBS at incubator for 24 h. Different concentrations of E9R were added into palate and incubated for 1 h, and then washed three times with PBS (pH 7.4). 4 μm of CS2‐N‐E9R was then added into each well and incubated for another 40 min. After that the samples were washed and imaged with Odyssey® Infrared Imager. Each data point represents the average value of quadruple wells. Data were analyzed using graphpad prism (GraphPad Software, San Diego, CA, USA). IC_50_ values, which were defined as the concentration of competitive agent required to inhibit 50% of CS2‐N‐E9R binding, were calculated.

E/F‐positive ES cells (A673) and E/F‐negative sarcoma cells (143B) were then seeded into confocal culture dishes at a density of 2 × 10^5^ cells each dish in 1.5‐mL medium containing 10% FBS. After 24 h of cell attachment, the dishes were then washed three times with 1 mL PBS. The dishes were then cultured in medium with 10 μm probe CS2‐N‐E9R. The intracellular probe aggregations were then observed by Leica‐LCS‐SP8‐STED confocal microscope (Leica, Wetzlar, Germany).

### 2.9 EWS–FLI1 targeted imaging of living cells

E/F‐positive ES cells (A673 cells, RD‐ES cells) and E/F‐negative osteosarcoma cells (143B cells), hepatoma carcinoma cells (Hep3B cells), and primary embryonic human kidney cells (293T cells) were seeded into confocal culture dishes. All the dishes were incubated with 10 μm CS2‐N‐E9R for 1 h and washed three times with PBS buffer before imaging, except for the control and blocking groups. For the control group, A673 cells were incubated with 10 μm control probe CS2‐N‐E. For the blocking group, A673 cells were pre‐incubated with 50 μm peptide E9R as a blocking agent for 1 h, after that incubated with 10 μm probe CS2‐N‐E9R.

### 2.10 Establishment of ES xenograft model and *in vivo* imaging

All procedures were carried out in compliance with the guide for the care and use of laboratory animal resources and the national research council, and approved by the Animal Biosafety Level 3 Laboratory of Wuhan University Institutional Animal Care and Use Committee (2019131). Mice were purchased from the Model Animal Laboratory of Wuhan University Zhongnan Hospital and housed in a specific pathogen‐free animal facility with a controlled environment (12 h light/dark cycle at 21 °C). To establish an ES xenograft mice model, A673 cells were grown subcutaneously around the right limb of 12‐16 g, 4‐week‐old Balb/c nude mice. Twenty‐one days after inoculation, the xenograft tumor mice were randomly divided into 3 groups (Experimental group/ CS2‐N‐E9R; Control group/ CS2‐N‐E; Blocking group/ CS2‐N‐E9R+E9R). Probes were freshly prepared at the concentration of 25 μm. The experimental group was given 150 μL targeted probe CS2‐N‐E9R, and the control group was given 150 μL nontargeted probe CS2‐N‐E through caudal vein injection. The blocking group was intratumoral pre‐injected 25 μL 25 μm E9R and incubated for 1 h before intravenously injection of 150 μL CS2‐N‐E9R. The imaging was carried out on Bruker Xtreme BI living small animals imaging system with an exaction laser of 690‐ and 750‐nm emission filter. Six hours after injection, one of the experimental group mice was sacrificed and the tumor tissue was resected and saved in dark environment for further immunofluorescence assay.

### 2.11 *In vivo* biodistribution and biocompatibility assays

At the following time points after CS2‐N‐E9R injection, 1, 6, 12 h, one of the ES‐bearing nude mice was sacrificed, and then, important organs and tissues were collected for *ex vivo* imaging detected by Bruker Xtreme BI. After that, the samples were fixed over 24 h in 4% paraformaldehyde and further embedded and sliced for fluorescence analysis by Leica‐LCS‐SP8‐STED confocal microscope.

The biocapacity assays carried out by H&E staining of vital organs including cerebrum, heart, liver, spleen, lung, stomach, colon, kidney, muscles, and bones showed that no obvious acute or chronic tissue damages on the tissue slices. The acute toxicity test was performed on 24 h after CS2‐N‐E9R intravenous injection, and the chronic toxicity test performed on 14 days of continuous intravenous injection every 2 days. Another Balb/c nude mouse was also sacrificed 24 h after saline injection as a control.

### 2.12 Establishment of orthotopic ES model and *in vivo* imaging

Balb/c nude mice were intratibially injected with 10^7^ A673 cells in 50 µl of PBS/50% Matrigel. Primary tumor development was examined weekly by activity of knee joint, X‐ray, and caliper measurements. Four weeks after tumor cells injection, when the primary tumor became detectable by X‐ray, the model mice were given 150 μL targeted probe CS2‐N‐E9R at concentration of 25 μm. The *in vivo* imaging was carried out on Bruker Xtreme BI imaging system 1 h after injection.

### 2.13 Establishment of ES lymph node metastasis and lymphadenitis in MRL/MpJ mice and *in vivo* imaging

Ewing sarcoma lymph node metastasis (LNM) mice model was established according to the previously reported method on MRL/MpJ mice [36]. We injected A673 cells into the right inguinal lymph node of MRL/MpJ mice. Twenty‐one days after injection, one of the experimental mice was sacrificed and its right inguinal lymph node was removed with the surrounding fat pad, and a pathology analysis was carried out to confirm the LNM mice model was accomplished. For the lymphadenitis mice model, we injected Freund's adjuvant into the right inguinal region of MRL/MpJ mice weekly for 2 weeks. Twenty‐one days after the first injection, pathology analysis was used to confirm the lymphadenitis mice model. The grouping of this experiment is according to the different mice models (LNM group; lymphadenitis group; CON group); after injection of 25 μL 25 μm CS2‐N‐E9R into the right footpad of the model mice, the imaging was carried out on Bruker Xtreme BI and settings as above.

### 2.14 Statistical analysis

To quantitate NIR fluoresces signal, a circular region‐of‐interest (ROI) was manually defined in the interested region, and the average signal within this ROI was obtained. Data were presented as mean ± SD. The statistical significance of differences was determined by ANOVA and LSD‐*t* test. A *P*‐value < 0.05 was considered to be significant: *P < 0.05, **P < 0.01, ***P < 0.001.

## 3 Result

### 3.1 The specificity of E9R toward EWS–FLI1 *in vitro*

To examine the *in vitro* interaction and binding affinity of peptide E9R toward E/F fusion protein, an *in vitro* pull‐down experiment was performed (Fig. 1B,C). To verify the specificity of E9R, a nonspecific 10 amino‐acid peptide E (QSSSYGQQND) was used for comparison. The protein pull‐down products were analyzed by western blot using anti‐Flag antibodies. Figure 1D showed the results of pull‐down experiment. As expected, biotin‐E9R was able to efficiently pull down Flag‐E/F. However, biotin‐E could not pull down the protein, indicating that biotin‐E does not bind to Flag‐E/F *in vitro*. Further, E9R did not bind to any proteins in the pull‐down eluates from control cells. In addition, we used anti‐EWSR1, anti‐FLI1, and anti‐Flag antibodies to detect biotin‐E9R pull‐down products. The results showed that anti‐EWSR1 antibody barely detect the Flag‐E/F but EWSR1. By contrast, both anti‐FLI1 and anti‐Flag antibodies detected the Flag‐E/F fusion protein in E9R pull‐down products (Fig. 1E). Overall, our results showed that E9R can selectively interact with E/F.

### 3.2 Characterization of CS2‐N‐E9R

Based on the aforementioned experimental results, we designed a probe (CS2‐N‐E9R) by linking a NIR cyanine dye CS‐2 with good photostability, high brightness, and sufficient chemical stability, to the amino side of E9R (Fig. 1A), and its corresponding probe (CS2‐N‐E) was also designed as a control. All the compounds were synthesized and fully characterized. The optical performance of CS2‐N‐E9R was then evaluated in PBS buffer (pH 7.4). The absorption and emission peaks occurred in NIR region at 718 and 748 nm, respectively (Fig. S10). CS2‐N‐E9R exhibited excellent photostability under physiological conditions, as proven by a slight fluorescence decrease about 4.8% after 2‐h irradiation at 690 nm (Fig. S11A). With insensitive to the variation of pH, CS2‐N‐E9R excited negligible florescence changes at the pH range from 3 to 10 (Fig. S11B). In addition, CS2‐N‐E9R showed good selectivity as many biologically relevant species including a variety of metal ions, anions, and amino acids have little influence on the fluorescence of the probe (Fig. S11C). Next, the cytotoxicity was tested by measuring the cell viabilities after culturing various concentrations of CS2‐N‐E9R with five types of cells including E/F‐positive ES cells (A673, RD‐ES), and E/F‐negative cells such as osteosarcoma cells (143B), hepatoma carcinoma cells (Hep3B), and primary embryonic human kidney cells (293T). As shown in Fig. S12, CS2‐N‐E9R displayed negligible or slight cytotoxicity to all the above cells even at concentration up to 20 μm. Altogether, the merits like good photostability, pH endurance, selectivity, and low cytotoxicity ensure CS2‐N‐E9R as a good NIR probe for ES bioimaging.

### 3.3 Colocalization of EWS–FLI1 with CS2‐N‐E9R in ES cells

**Fig. 2:**
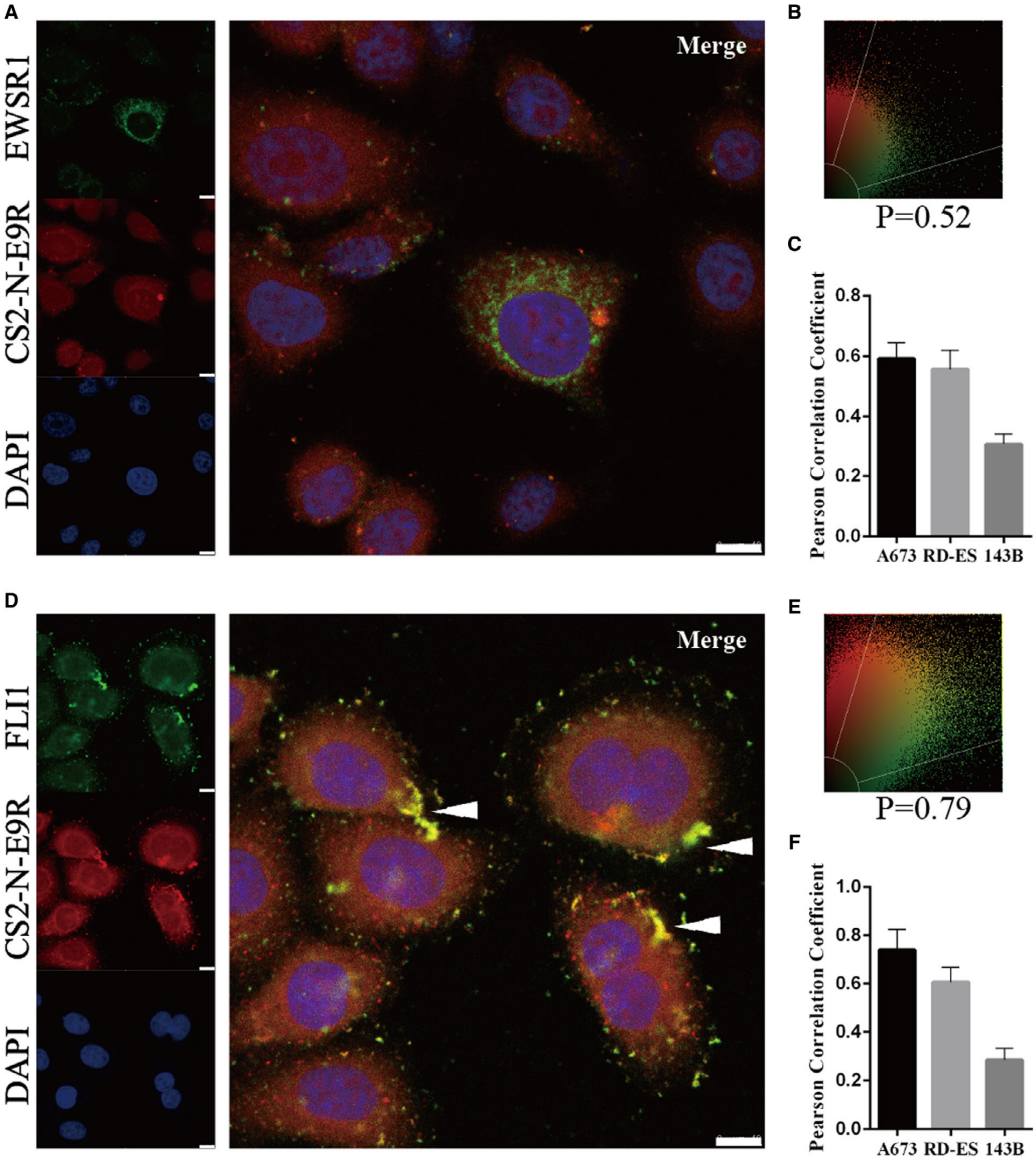
Colocalization of E/F with CS2‐N‐E9R in A673 cells (*n* = 3 for each cell). The blue channel of the DAPI dye was collected at 360–400 nm. The green channel indicates EWSR1 (A) or FLI1 (D) collected at 500–540 nm (the white arrowhead shows the cells undergoing division). The red channel of probe CS2‐N‐E9R was collected at 750 nm with excitation at 633 nm. Scale bar = 10 μm. Colocalization of EWSR1 (B) or FLI1 (E) with CS2‐N‐E9R assessed by Pearson Correlation Coefficient. (C, F) The differences between A673 cells, RD‐ES cells and 143B cells in colocalization coefficient as shown in the bar graph (*n* = 3, mean ± SD).

To further investigate the binding and subcellular localization of CS2‐N‐E9R, the colocalization experience was carried out. Here, E/F was labelled with commercial antibodies against EWSR1 and FLI1 using immunofluorescence cytochemistry. A673 and RD‐ES cells were costained by CS2‐N‐E9R and two types of commercial antibodies, respectively. 143B cells were also subjected to the above experiments as a control sarcoma cell (Fig. S13). Figure 2A showed that the green channel (anti‐EWSR1) could not effectively label ES cells, and the colocalization rates for EWSR1 and CS2‐N‐E9R were not high with Pearson Correlation Coefficient at 0.52 (Fig. 2B). In Fig. 2D, the green channel (anti‐FLI1) and the red channel (CS2‐N‐E9R) overlayed nicely, and the overlap Pearson Correlation Coefficient reached at 0.79 (Fig. 2E). Moreover, it could be seen that the expression of E/F and the colocalization degree between CS2‐N‐E9R and E/F in ES cells undergoing division were much higher than other cells (Fig. 2D, showed by white arrow), which was related to the role of E/F in ES cell proliferation. However, the correlation coefficients of the above experimental results in 143B cells were < 0.5, which could not prove the colocalization relationship between the above‐mentioned proteins antibodies and CS2‐N‐E9R (Fig. 2C,F). These findings further verified that CS2‐N‐E9R had the binding capacity toward ES cells at the cellular level.

### 3.4 Selective imaging of living ES cells

**Fig. 3:**
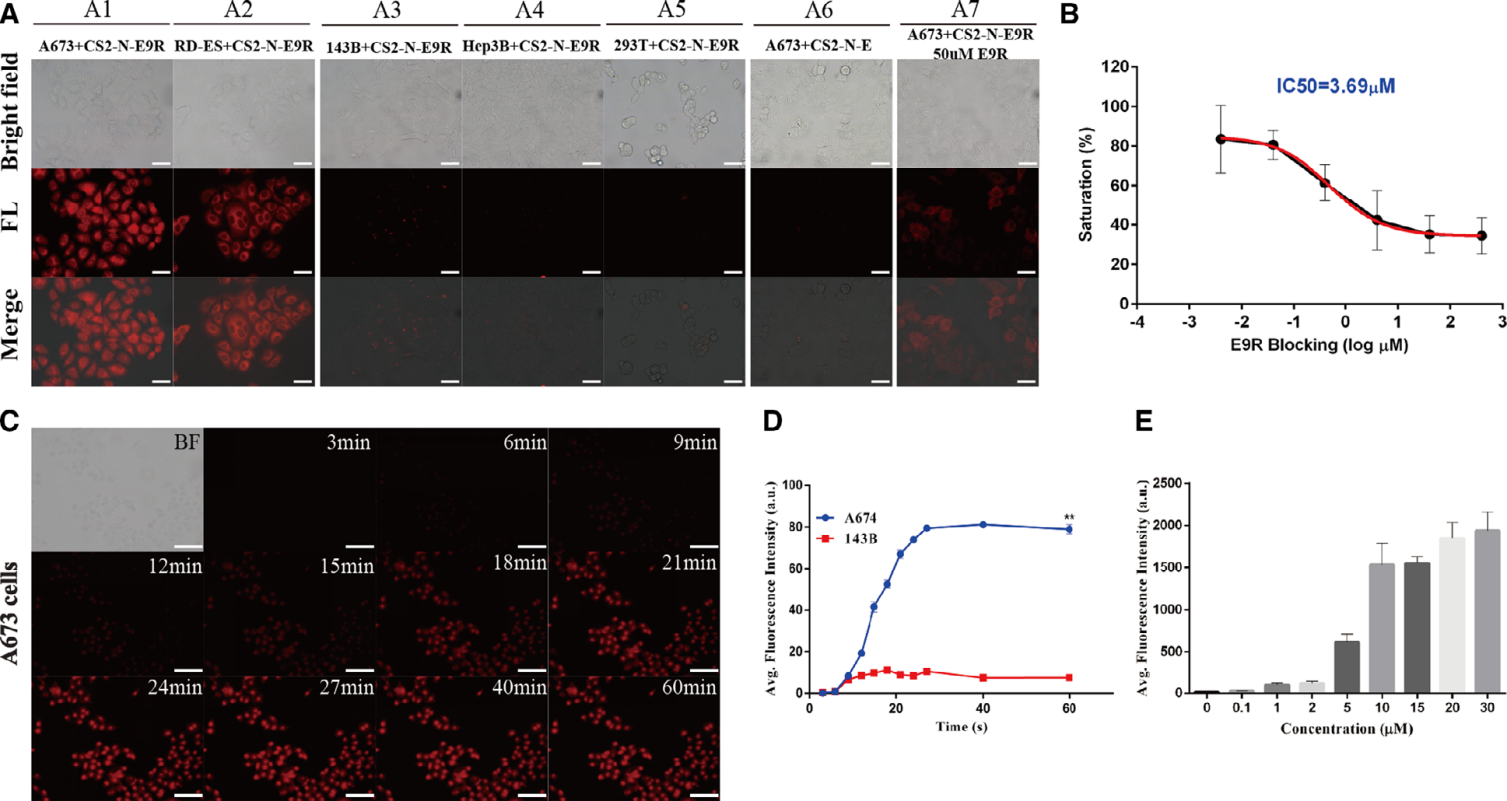
Selective imaging of living ES cells (*n* = 3 for each cell). (A) The fluorescence imaging of A673 cells, RD‐ES cells, 143B cells, Hep3B cells or 293T cells incubated with probes for 1 h. Scale bar = 50 μm. (B) Binding affinity of CS2‐N‐E9R to A673 cells using E9R as the blocking agent. (C) Time responses of 10 μm CS2‐N‐E9R in living A673 cells. Scale bar = 100 μm. (D) Fluorescence emission intensities of red channel were measured as averages of 4 regions of interest (ROIs) from different time points in A673 cells and 143B cells (Student's *t*‐test, **P = 0.0011). (E) Intracellular fluorescence intensity of A673 when incubated with various concentration of CS2‐N‐E9R. Data are expressed as mean ± SD.

We next evaluated the imaging ability of CS2‐N‐E9R in living cells by a set of assays. First, we compared the intracellular fluorescence between the E/F‐positive ES cells A673, RD‐ES, and the E/F‐negative cells such as, 143B, Hep3B, and 293T. As shown in Fig. 3A, A673 cells and RD‐ES cells (Fig. 3A‐A1,A2) exhibited strong intracellular fluorescence, while E/F‐negative cells displayed negligible signals in the presence at 10 μm CS2‐N‐E9R (Fig. 3A‐A3–A5), indicating CS2‐N‐E9R realized precise imaging of ES cells and separated it from other cells as well. Meanwhile, a control assay using the nonspecific probe CS2‐N‐E performed in A673 cells showed extremely weak fluorescent signals (Fig. 3A‐A6), and it is again confirmed that CS2‐N‐E could not target ES cells and could be used as a control probe. In an additional competitive binding assay, the fluorescence muted when A673 cells were pretreated with 50 μm E9R as an inhibitor before incubation with CS2‐N‐E9R (Fig. 3A‐A7). The results of this comparison are presented in Fig. S14. Subsequently, we used E9R as a blocking agent and incubated the probe with ES cells. The results showed that E9R can effectively block the binding of the probe to E/F, suggesting that E9R may have a space occupancy and blocking effect of the binding site (Fig. 3A‐A7).

Furthermore, we performed a binding affinity test to verify the above conjecture, and the IC_50_ value for the inhibition of CS2‐N‐E9R binding E/F in A673 cells was calculated to be 3.69 μm (Fig. 3B). The following time‐dependent living cells imaging (Fig. 3C) showed a gradually fluorescence increasing and reached a plateau at around 24 min in a 1‐h assay, while negligible fluorescent signal in 143B cells was observed at the same time points (Fig. S15). Figure 3D was a quantitative data collection of intracellular fluorescence intensity, suggesting the specific uptake of CS2‐N‐E9R by A673 cells, and no continuous uptake in non‐ES cells was observed. Finally, we also set up different concentrations of probes to co‐incubate with A673 cells. The concentration of CS2‐N‐E9R uptake by A673 cells tends to be saturated at about 10 μm, and based on that, we set the probe concentration for *in vitro* and *in vivo* experiments (Fig. 3E).

### 3.5 *In vivo* and *ex vivo* imaging ES lesions and determination of tumor boundaries

**Fig. 4:**
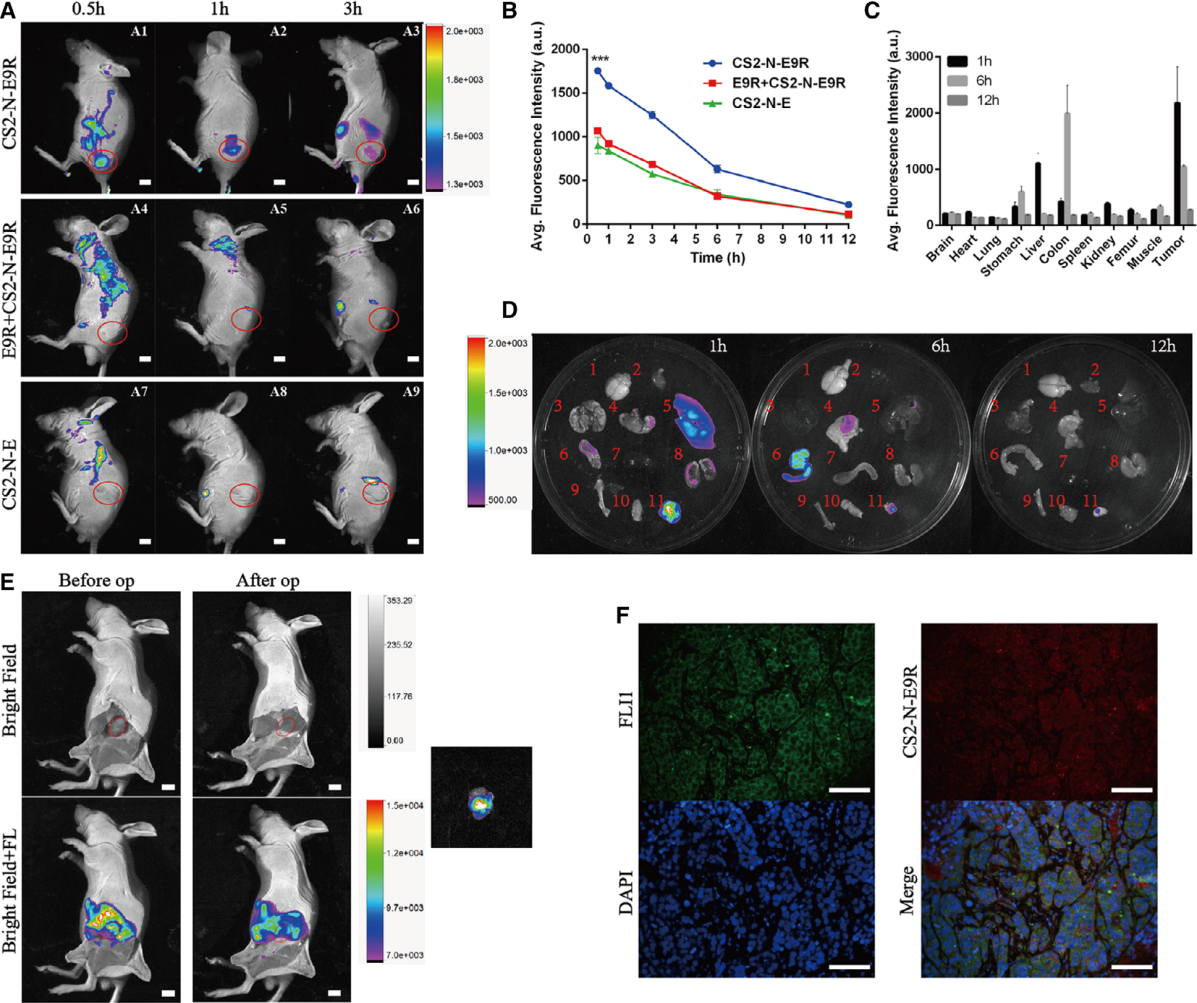
*In vivo* live imaging of mice with subcutaneously grafted tumor. (A) *In vivo* NIR fluorescence imaging of ES in Balb/c‐nude mice bearing A673 xenograft tumors (*n* = 3 mice for each group). Scale bar 4 mm. (B) Fluorescence emission intensities of tumor regions were measured as averages of four ROIs from different groups (*n* = 3, statistically significant differences were determined with ANOVA test for multiple comparisons and Student's *t*‐test when comparing two groups, ***P < 0.001). (C) Fluorescence intensities of tissues at different time points (1, 6, 12) after probe injection were collected and measured as average of four regions of interest (ROIs). (D) The biodistribution of CS2‐N‐E9R at different time points (1, 6, 12 h). 1: cerebrum; 2: heart; 3: lung; 4: stomach; 5: liver; 6: colon; 7: spleen; 8: kidney; 9: bone; 10: muscle; 11: tumor (*n* = 3 for each group). (E) The tumor boundary indicated by CS2‐N‐E9R during operation (op). *n* = 3 mice. (F) Immunofluorescence assay of tumor tissues (*n* = 3). Scale bar = 100 μm. Data are expressed as mean ± SD.

After confirming the specificity of CS2‐N‐E9R at the cellular level, we were encouraged to investigate the *in vivo* application of CS2‐N‐E9R via ES‐bearing mice model (Fig. S16). Figure 4A‐A1 and showed that CS2‐N‐E9R rapidly accumulated in the tumor region (marked by red circles); after injection, the fluorescence intensity of the tumor region was about 1.6‐fold stronger than the background fluorescence of the animal body. The fluorescence of the tumor region of the experimental group remained visible at 3 h (Fig. 4A‐A3) in real‐time *in vivo* imaging and then gradually faded. On the contrary, in the control group and the blocking group (locally incubated with 50 μm blocking agent), no fluorescence higher than the background was captured (Fig. 4A‐A4,A5). Figure 4B quantitatively showed the changes in fluorescence intensity of the tumor region over time during *in vivo* imaging. The *ex vivo* biodistribution studies (Fig. 4C,D) demonstrated the distribution of probes in important organs and tissues in mice model by fluorescence imaging. The results showed that the probe mainly metabolized by the liver (1 h; 5) and excreted by the intestine (6 h; 6), and also, it did not accumulate in important organs and tissues, demonstrating a rapid clearance of CS2‐N‐E9R from the system. Only a weak fluorescence signal could be seen in the tumor tissue 12 h after injection, while other tissues have no fluorescence showed, which was in accordance with the additional confocal images of tissue slices (Fig. S17). Notably, Fig. 4E indicated a strong evidence of the ability of CS2‐N‐E9R to accurately enhance the signal of tumor region (the red dotted line indicated the tumor boundaries) for the identification of tumor boundaries and isolate it from background *in vivo* at 1 h after injection. The excised tumor tissue was labeled with anti‐FLI1 antibody to confirm that the probe was molecularly targeted to E/F and thus labeled the tumor tissue (Fig. 4F). Afterward, we also performed biocompatibility assays (Fig. S18) to show that the probe did not cause acute or chronic damage to important organs or tissues.

### 3.6 *In vivo* and *ex vivo* imaging in an orthotopic ES mouse model

**Fig. 5:**
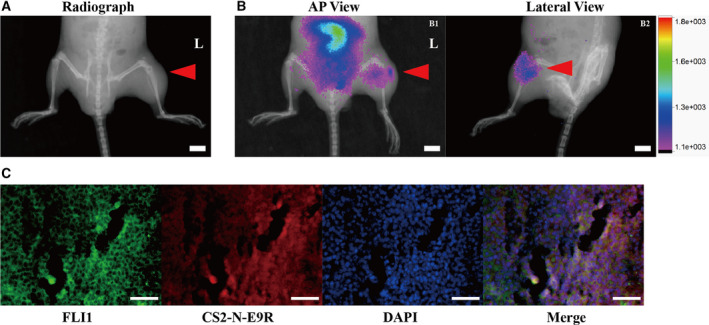
*In vivo* live imaging of mice with orthotopic tumor. (A) X‐ray imaging and (B) merge of X‐ray and NIR imaging of orthotopic tumor (Left) in the nude mouse (*n* = 3 mice). The red arrowhead indicated the orthotopic tumor. Scale bar = 4 mm. (C) Immunofluorescence assay of orthotopic tumor tissues (*n* = 3). Scale bar = 100 μm.

To investigate the potential of using probe CS2‐N‐E9R to detect orthotopic ES tumor, we carried out noninvasive NIR *in vivo* imaging in orthotopic mice model (Fig. S19). As shown in Fig. 5A, X‐ray of the left knee shows a swollen joint and bony destruction; however, the tumor boundaries cannot be clearly displayed by small animal X‐ray (Fig. 5A). The Gross morphology of the tumor was clearly observed from 1 h postinjection at both anteroposterior (AP) view and lateral view (Fig. 5B). *Ex vivo* tumors were further confirmed by H&E staining (Fig. S19) and immunofluorescence staining (Fig. 5C), once again demonstrating that the E9R probe can be used to accurately image ES by targeting E/F protein.

### 3.7 Intraoperative fluorescence intervention for detection of lymph node metastases

**Fig. 6:**
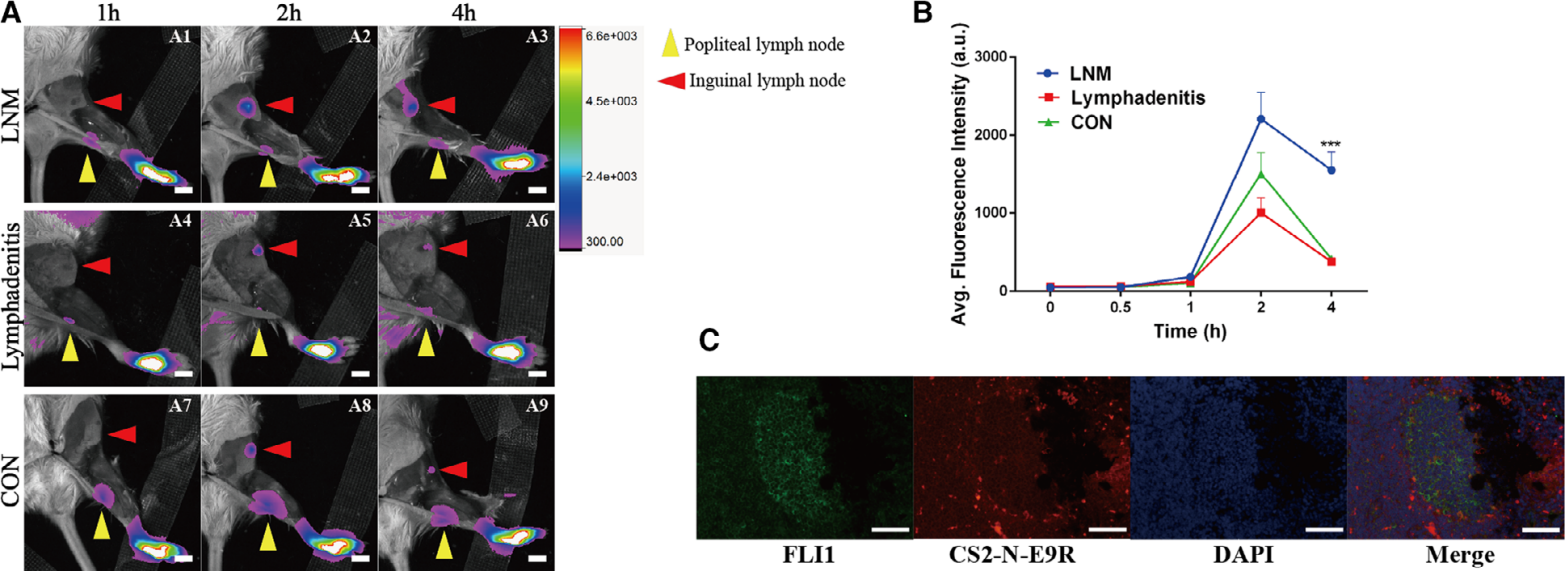
*In vivo* live imaging of mice with LNM. (A) *In vivo* NIR fluorescence imaging in LNM mice model, lymphadenitis mice model, and normal MRL/MpJ mice (yellow arrowhead shows popliteal lymph node; red arrowhead shows inguinal lymph node). *n* = 3. Scale bar = 4 mm. (B) Fluorescence emission intensities of the inguinal lymph node regions were measured as averages of four regions of interest (ROIs) from different groups (*n* = 3, statistically significant differences were determined with ANOVA test for multiple comparisons and Student's *t*‐test when comparing two groups, ***P < 0.001). (C) Immunofluorescence assay of tumor metastatic popliteal lymph node (*n* = 3). Scale bar = 100 μm. Data are expressed as mean ± SD.

Considering the excellent performances on targeting E/F and imaging ES tumor in mice, we thought that CS2‐N‐E9R might also be able to detect ES LNM as well. Therefore, we constructed the LNM mouse model (Fig. S20). Figure 6A provided a comprehensive understanding of the real‐time process of CS2‐N‐E9R draining from the lymphatic vessel network under mice model footpad through the popliteal lymph node (showed by yellow arrow) to the inguinal lymph node (showed by red arrow). The probe began to gradually accumulate in the inguinal lymph node area 1 h after injection in the experimental group (Fig. 6A‐A1), reached a peak at 2 h (Fig. 6A‐A2), and then began to decline, and 4 h after injection (Fig. 6A‐A3), the fluorescence intensity of the inguinal lymph nodes remained 3.7‐fold stronger compared with the popliteal lymph nodes, and quantitative data statistics were shown in Fig. 6B. However, the fluorescence intensity of inflammation group (Fig. 6A‐A6) and control group (Fig. 6A‐A9) decreased to the same as the popliteal lymph nodes. The above results suggested that CS2‐N‐E9R was up taken by ES cells in the LNM and retained in the lymph node, which was helpful for detecting the LNM during the operation and distinguishing the metastasis from the lymph nodes enlarged due to sterile inflammation. Again, further immunofluorescence assay confirmed that CS2‐N‐E9R achieved LNM recognition by targeting E/F (Fig. 6C).

## 4 Discussion

E/F is a crucial fusion protein in ES that governs many transcription and translation processes, thereby affecting tumor initiation and progression [37]. It is considered to be a promising target for the treatments of ES [33]. However, since E/F is an intrinsically disordered protein, the research on the structure and function of this targeting protein had been hindered and the development of new diagnosis and treatment methods for ES had been restricted [38]. In this study, we presented the first ES‐specific probe CS2‐N‐E9R, which offers satisfying stability and sensitivity for real‐time *in vitro* and *in vivo* E/F detection and we believed the feasibility of using the E/F‐targeting fluorescein‐labeled peptide E9R for experimental research on ES. We also expect that this study will enable other researchers to obtain a wider range of tools for the research of RHA and E/F binding targets.

It is worth noting that several difficulties in the clinical diagnosis and treatment of ES might be solved through ES‐targeted probe CS2‐N‐E9R. First of all, there is no noninvasive imaging method for the diagnosis of ES at present [39]. Since sarcomas have similar characteristics and the clinical manifestations of sarcomas are not characteristic, it is difficult to distinguish ES from other bone and soft tissue sarcomas through CIMs [40]. However, CS2‐N‐E9R can adopt real‐time fluorescence imaging in the NIR region to achieve noninvasive imaging and realize ES diagnosis. Secondly, there currently lack of tools to specifically identify molecular subtypes of sarcomas, which affects the evaluation of prognostic differences between patients with expressing different genotypes [41]. Histopathology remains the gold standard for ES diagnosis, which can only be administered by experienced pathologist to diagnose E/F‐positive ES [42]. Our *in vitro* experiments confirmed the specificity of targeting peptide E9R has the ability to bind E/F, which provides a viable alternative for sarcoma genotyping, negating the need for invasive tumor tissue biopsy.

Moreover, surgical resection remains the first choice in terms of ES treatment, since researches on innovative drugs for ES have almost no progress [4]. In order to prevent relapse and ensure prognosis, patients usually treated with expand resection with an additional cuff of normal tissue and regional lymph nodes dissection. This kind of surgery will bring consequences to patients, such as skin or soft tissue defects caused by excessive resection, and a partial resection of bone when the tumor invades the periosteum which may cause bone defects, nonunion, shortened extremities, or even amputation [43]. The usual measures of complete regional lymph nodes dissection, which will cause the impairment of lymph flowback, and in severe cases may complicated with perioperative bleeding or lymphedema [44]. Adequate surgical boundary significantly affects the outcome for patients with ES [45]. Therefore, it is particularly important to develop a real‐time display of the tumor boundaries during surgery to guide the precise section of tumor tissues. In our *in vivo* studies, we verified the CS2‐N‐E9R can realize intraoperative fluorescence intervention via rapid imaging and ES tissue retention capability in order to distinguish between metastatic lymph nodes and lymphadenitis, and we used MRL/Mpj model mice for *in vivo* experiments and verified that CS2‐N‐E9R can indicate lymph node drainage and arrest in metastatic lymph nodes for reaction guidance of early‐stage lymph node micrometastases.

Adjuvant and neoadjuvant chemotherapy as an adjunct to radical surgery has a favorable outcome of surgical treatment and prognostic effect on patients with ES [46]. A targeted imaging approach for ES would also help alleviate the diagnostic quandary posed by the early stage of micrometastatic lesions using CIMs, both at the time during post‐treatment surveillance. Both adjuvant and neoadjuvant chemotherapies have the limitation that it cannot achieve killing cancer cells without damaging normal tissue cells. Therefore, the E/F molecular targeted ES probe may allow repetitive assessment of the *in vivo* accumulation and targeted drug release may facilitate studies to guide precise medication of chemotherapy drugs for patients in the future. CS2‐N‐E9R also has the ability to monitor the lesions under chemotherapy in real time, which plays a significant role in assessing the response of patients to therapy.

Our study also has certain limitations. We designed a small molecule peptide probe, although this set of probes has low immunogenicity and good cell penetration ability to achieve intracellular imaging and is an ideal diagnostic and therapeutic drug components, and it has the disadvantage of being catabolized by proteases fast in the system, which is not conducive to long‐term real‐time living imaging. Secondly, the fluorophore we selected has good cell permeability, stability, and low toxicity, but the background ratio for *in vivo* imaging is not high enough. However, we expect that the above limitations will be circumvented in the near future.

## 5 Conclusions

In conclusion, we have developed an ES‐specific probe, namely, CS2‐N‐E9R, with high sensitivity and selectivity toward E/F fusion protein. CS2‐N‐E9R can expeditiously differentiate ES cells from normal cells. By molecularly targeting E/F, CS2‐N‐E9R shows promise for differential diagnosis of sarcoma subtypes and accurate identification of intraoperative tumor borders as well as LNM. Our findings also suggest that CS2‐N‐E9R could be used for preoperative visualizing to reduce the damage to normal tissues and improve therapeutic outcomes. Through real‐time imaging of ES, CS2‐N‐E9R may serve as a useful tool for the development of new chemotherapeutic drugs and offering image‐guided interventions, including resections and monitoring of efficacy.

## Conflict of interest

The authors declare no conflict of interest.

## Author contributions

YW, XH, and AY conceptualized the data; YW, HM, and XH performed data curation; YW, SW, XH, and AY involved in formal analysis; SW, XH, and AY contributed to funding acquisition; YW, HM, YY, and HC investigated the research; YW and HM performed software; SW, XH, and AY supervised the manuscript; YW, HM, SW, XH, and AY validated the document; YW involved in visualization; YW performed roles/wrote original draft; YW, HM, SW, XH, and AY reviewed and edited the document.

### Peer review

The peer review history for this article is available at https://publons.com/publon/10.1002/1878‐0261.13081.

## Supporting information


**Fig. S1**. ^1^H NMR spectrum of **CS‐2** in CDCL_3_ solution.
**Fig. S2**. HRMS of E9R.
**Fig. S3**. HPLC traces of E9R.
**Fig. S4**. HRMS of CS2‐N‐E9R.
**Fig. S5**. HPLC traces of CS2‐N‐E9R.
**Fig. S6**. HRMS of CS2‐N‐E.
**Fig. S7**. HPLC traces of CS2‐N‐E.
**Fig. S8**. Quantum yield measurements of CS2‐N‐E9R.
**Fig. S9**. Gel electrophoresis results for the verification of pCMV‐Flag‐E/F.
**Fig. S10**. UV‐Vis and fluorescence spectra results.
**Fig. S11**. The results of optical measurements.
**Fig. S12**. Cytotoxity of CS2‐N‐E9R through CCK‐8 assay.
**Fig. S13**. Co‐localization imaging of EWS‐FLI1 and CS2‐N‐E9R in RD‐ES cells and 143B cells.
**Fig. S14**. Quantification of live cell imaging analysis.
**Fig. S15**. Time responses of 10 μm probe CS2‐N‐E9R in living 143B cells.
**Fig. S16**. The establishment of ES xenograft model.
**Fig. S17**. Fluorescence imaging of tumor and normal tissues slices at different time points.
**Fig. S18**. The biocapacity assays was carried out by tissue slice histopathology analysis.
**Fig. S19**. The establishment of orthotopic ES model.
**Fig. S20**. The establishment of ES LNM and lymphadenitis in MRL/MpJ mice.

## Data Availability

The data sets used and/or analyzed during the current study are available from the corresponding author on reasonable request.
