# Supplementary material for: EWS–FLI1‐targeting peptide identifies Ewing sarcoma tumor boundaries and lymph node metastasis via near‐infrared imaging
Source: Mol Oncol. 2021 Aug 30;15(12):3706–20. doi: 10.1002/1878-0261.13081 (PMC8637573; doi:10.1002/1878-0261.13081)
Supplement: Supplementary file 1 — Fig. S1. 1H NMR spectrum of CS‐2 in CDCL3 solution. Fig. S2. HRMS of E9R. Fig. S3. HPLC traces of E9R. Fig. S4. HRMS of CS2‐N‐E9R. Fig. S5. HPLC traces of CS2‐N‐E9R. Fig. S6. HRMS of CS2‐N‐E. Fig. S7. HPLC traces of CS2‐N‐E. Fig. S8. Quantum yield measurements of CS2‐N‐E9R. Fig. S9. Gel electrophoresis results for the verification of pCMV‐Flag‐E/F. Fig. S10. UV‐Vis and fluorescence spectra results. Fig. S11. The results of optical measurements. Fig. S12. Cytotoxity of CS2‐N‐E9R through CCK‐8 assay. Fig. S13. Co‐localization imaging of EWS‐FLI1 and CS2‐N‐E9R in RD‐ES cells and 143B cells. Fig. S14. Quantification of live cell imaging analysis. Fig. S15. Time responses of 10 μm probe CS2‐N‐E9R in living 143B cells. Fig. S16. The establishment of ES xenograft model. Fig. S17. Fluorescence imaging of tumor and normal tissues slices at different time points. Fig. S18. The biocapacity assays was carried out by tissue slice histopathology analysis. Fig. S19. The establishment of orthotopic ES model. Fig. S20. The establishment of ES LNM and lymphadenitis in MRL/MpJ mice. [file MOL2-15-3706-s001.pdf]

Supplementary materials

EWS–FLI1-targeting peptide identifies Ewing sarcoma tumor boundaries and lymph node metastasis via near-infrared imaging

Yu Wang [a], Hengtang Mai [b], Ying Yuan [a], Hairen Chen [a], Song Wu [b], Xiang Hu [a,*] and Aixi Yu [a,*]

[a.] Department of Orthopaedic Trauma and Microsurgery, Wuhan University Zhongnan Hospital, 169 East Lake Road, Wuhan, Hubei, 430071, P. R. China

[b.] Hubei Province Engineering and Technology Research Center for Fluorinated Pharmaceuticals, School of Pharmaceutical Sciences, Wuhan University, 115 East Lake Road, Wuhan, Hubei, 430072, P. R. China

E-mail: shawnhu2002@whu.edu.cn; yuaixi@whu.edu.cn

Synthesis of CS-2: ^{1}H NMR (400 MHz, $CDCl_3$) δ 8.58 (d, J = 13.9 Hz, 1 H), 8.26 (d, J = 7.7 Hz, 1 H), 7.73 (t, J = 7.6 Hz, 1 H), 7.59 (t, J = 7.6 Hz, 1 H), 7.38-7.45 (m, 2 H), 7.16-7.25 (m, 3 H), 6.59-6.73 (m, 3 H), 6.06 (d, J = 13.2 Hz, 1 H), 3.68 (s, 3 H), 3.53 (q, 4 H), 2.67 (t, J = 6.1 Hz, 2 H), 2.25-2.35 (m, 2 H), 1.81 (d, J = 2.2 Hz, 8 H), 1.28 (t, J = 6.9 Hz, 6 H).

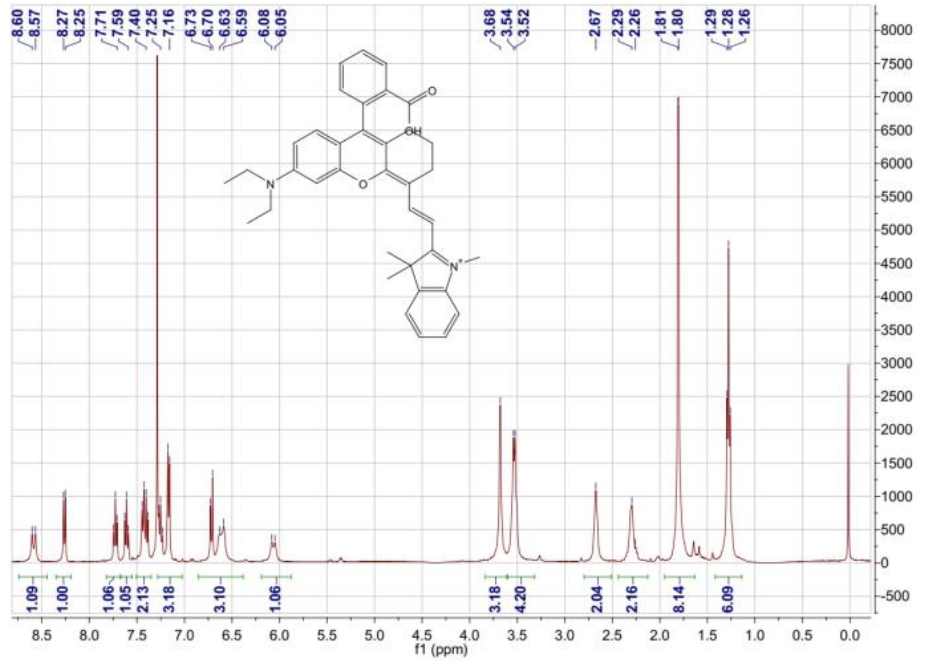

Fig. S1. ^{1}H NMR spectrum of CS-2 in $CDCL_3$ solution.

Synthesis of the peptide E9R:

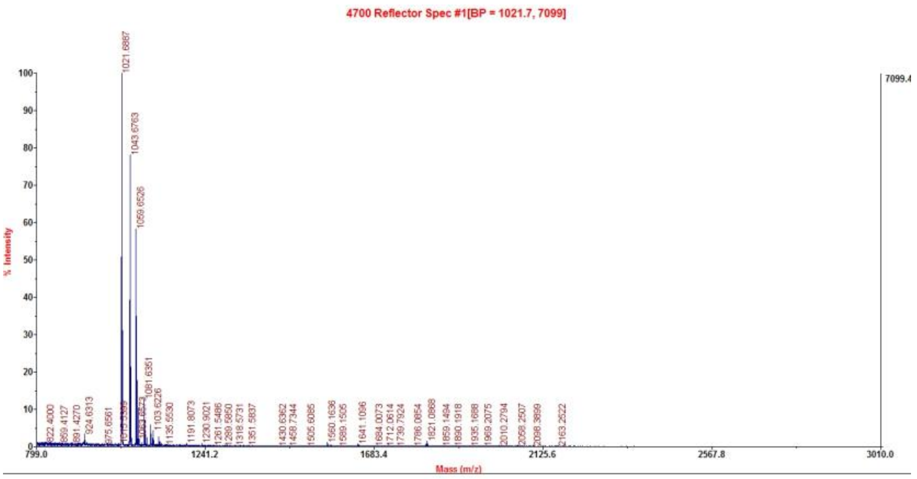

Fig. S2. HRMS of E9R. HRMS (ESI) for $C_{47}H_{76}N_{10}O_{15}$: calcd for $[M+H]^+$, 1021.5514; found, 1021.6887.

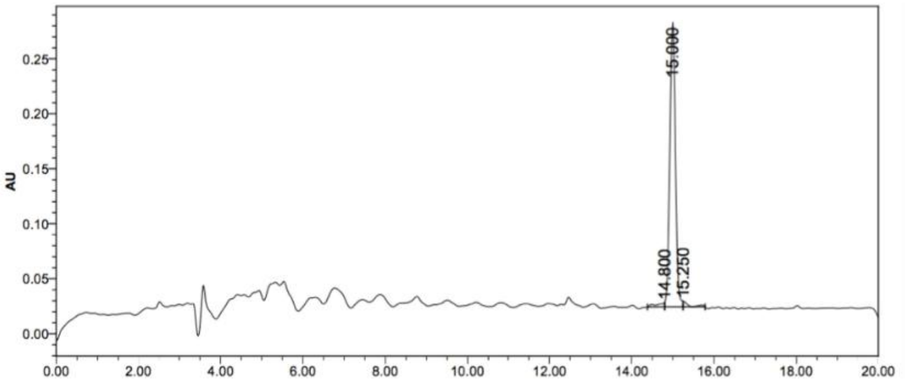

Fig. S3. HPLC traces of E9R.

Synthesis of the CS2-peptide:

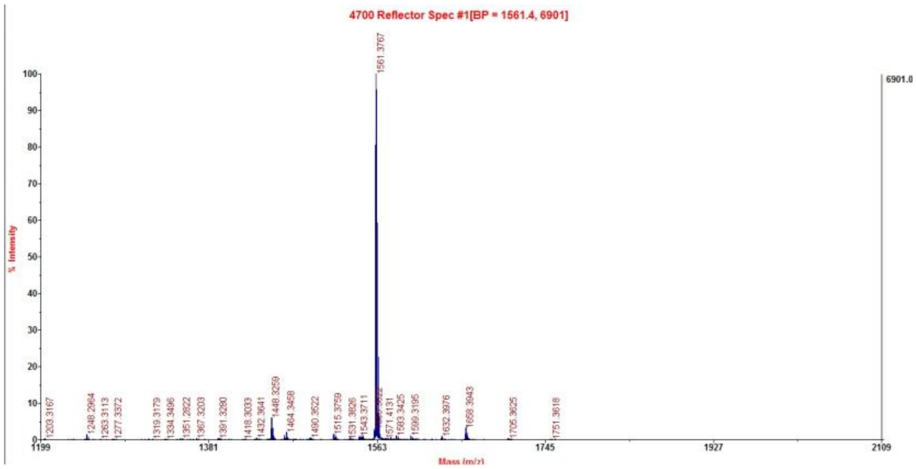

Fig. S4. HRMS of CS2-N-E9R. HRMS (ESI) for $C_{84}H_{113}N_{12}O_{17}^{+}$, ($[M+H]^{+}$): calcd 1561.8314, found 1561.3767.

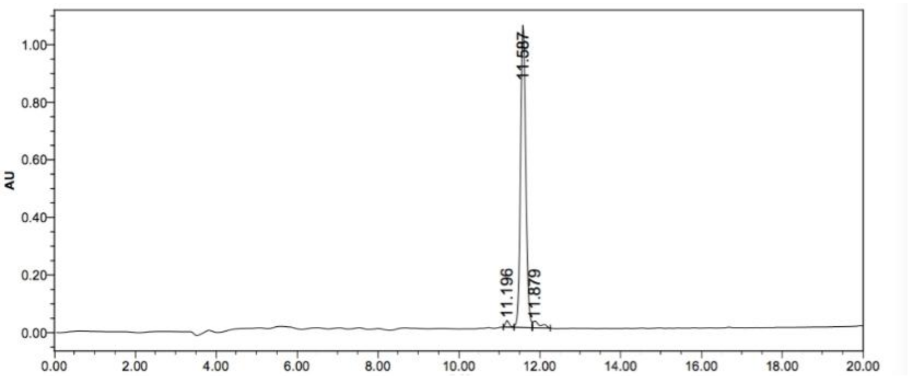

Fig. S5. HPLC traces of CS2-N-E9R.

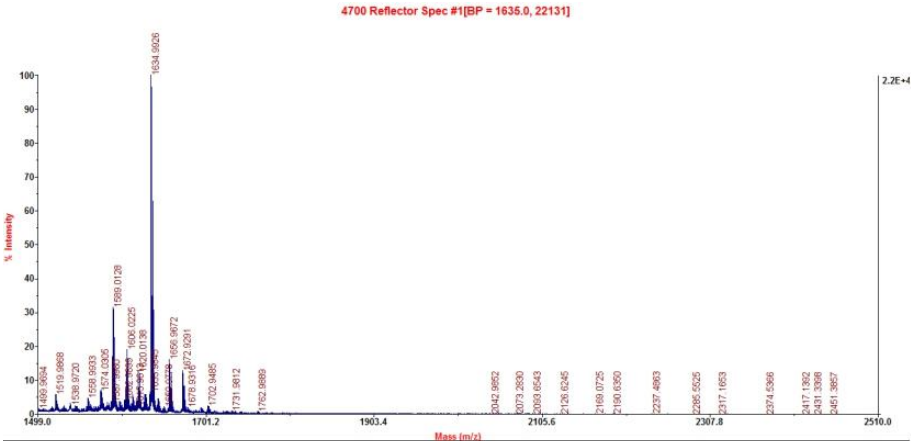

Fig. S6. HRMS of CS2-N-E. HRMS (ESI) for $C_{51}H_{103}N_{16}O_{21}^{+}$, $([M+H]^{+})$: calcd 1561.8314, found 1561.3767.

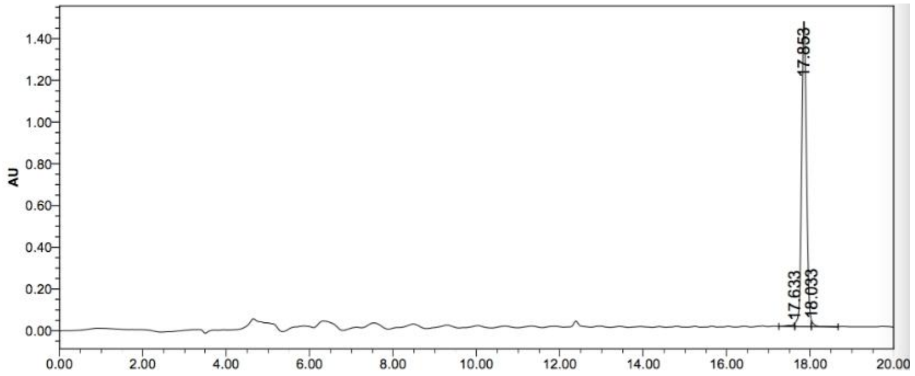

Fig. S7. HPLC traces of CS2-N-E.

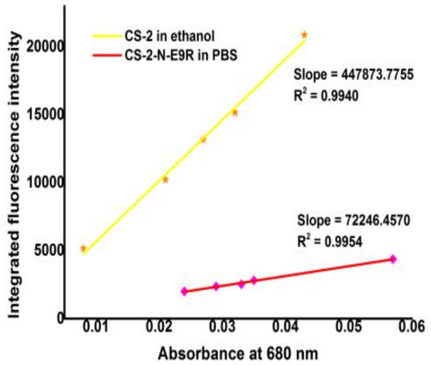

Fig. S8. Quantum yield measurements of CS2-N-E9R. CS-2 in ethanol was used as a standard. Five different concentrations were measured and the tendency chart of integrated fluorescence intensity versus absorbance was plotted.

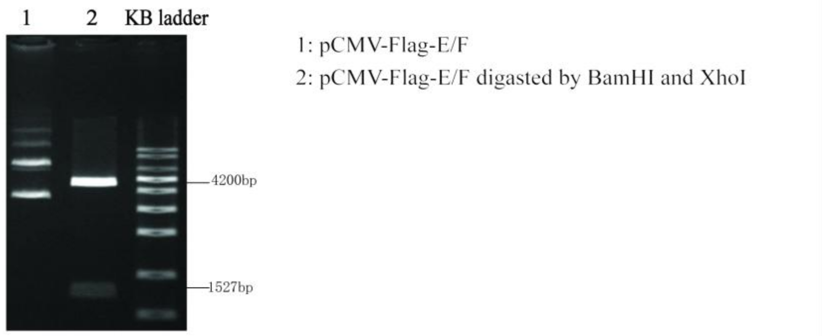

Fig. S9. Gel electrophoresis results for the verification of pCMV-Flag-E/F. About 300 ng plasmid was digested by enzyme in water-bath at 37 °C for 40 minutes.

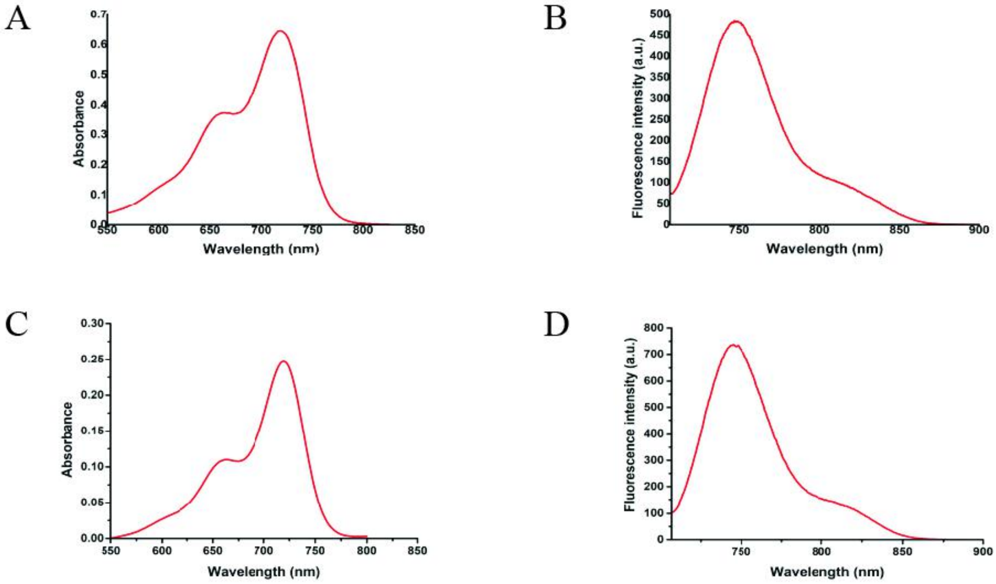

Fig. S10. UV-Vis and fluorescence spectra results. (A) UV-Vis and (B) fluorescence spectra of 10 μM CS2-N-E9R in PBS buffer (0.01 M, pH 7.4). (C) UV-Vis and (D) fluorescence spectra of 10 μM CS2-N-E at the same condition.

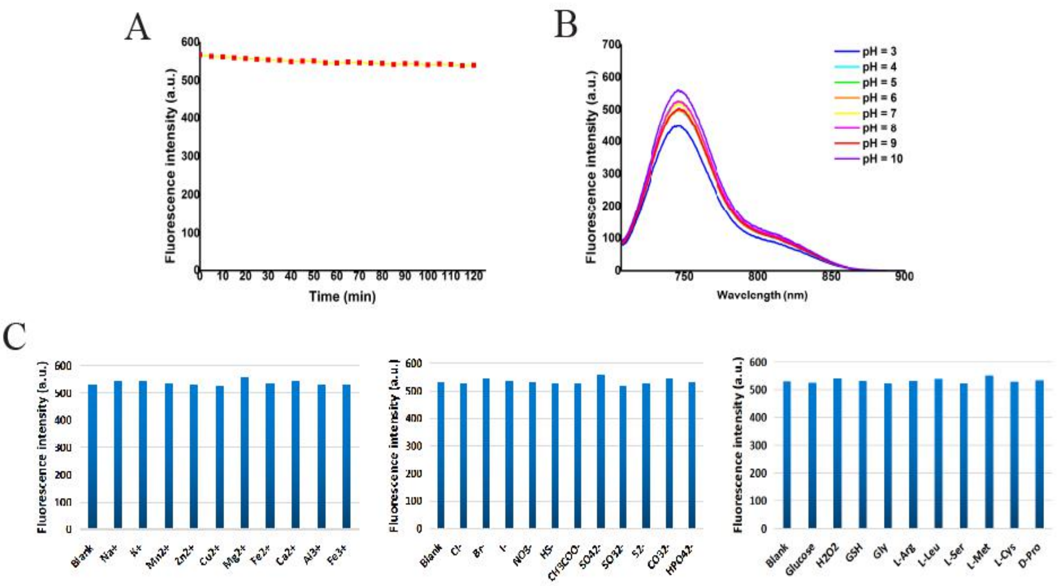

Fig. S11. The results of optical measurements. (A) Fluorescence intensity of 10 μM CS2-N-E9R versus excitation time in PBS buffer (0.01 M, pH 7.4). (B) Fluorescence intensity of 10 μM CS2-N-E9R in various pH solutions (range from 3-10). (C) Fluorescence responses of probe CS2-N-E9R (10 μM).

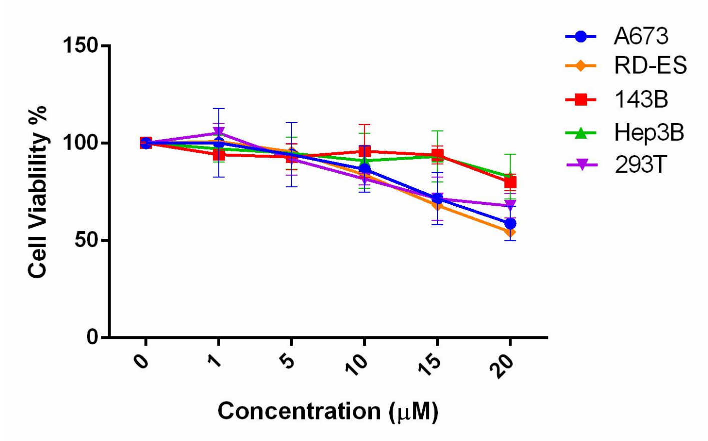

Fig. S12. Cytotoxity of CS2-N-E9R through CCK-8 assay. The cell viability related to the absorbance at 450 nm. Each data point represents an average of three wells. Data are expressed as mean ± SD.

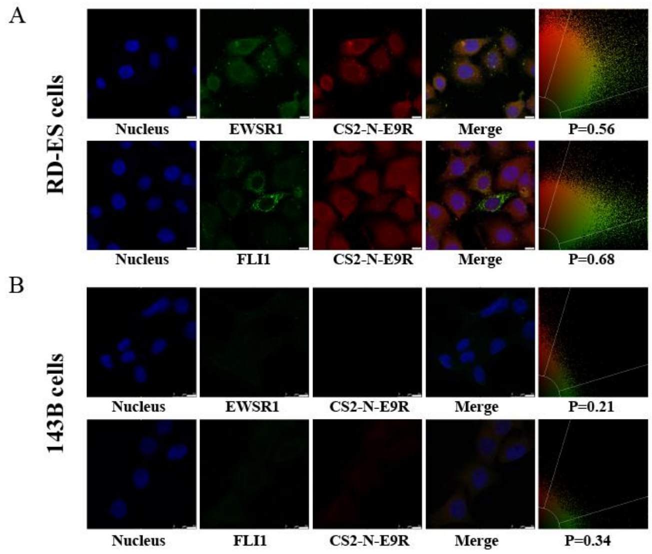

Fig. S13. Co-localization imaging of EWS-FLI1 and CS2-N-E9R in RD-ES cells and 143B cells. The blue channel of the DAPI dye was collected at 360-400 nm. The green channel indicates EWSR1 or FLI1 collected at 500–540 nm. The red channel of probe CS2-N-E9R was collected at 750 nm with excitation at 633 nm (n = 3 for each cell). Scale bar = 10 μm.

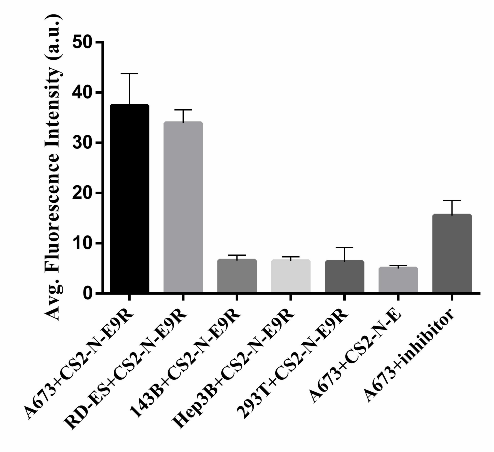

Fig. S14. Quantification of live cell imaging analysis. Fluorescence emission intensities were measured as averages of four ROIs from different groups (n = 3, mean ± SD).

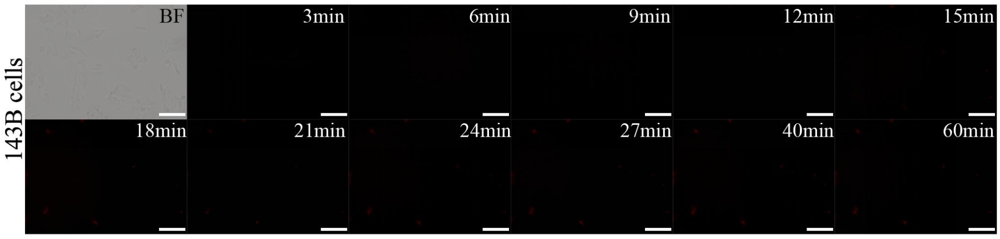

Fig. S15. Time responses of 10 μM probe CS2-N-E9R in living 143B cells. λex = 633 nm (n = 3). Scale bar =100 μm.

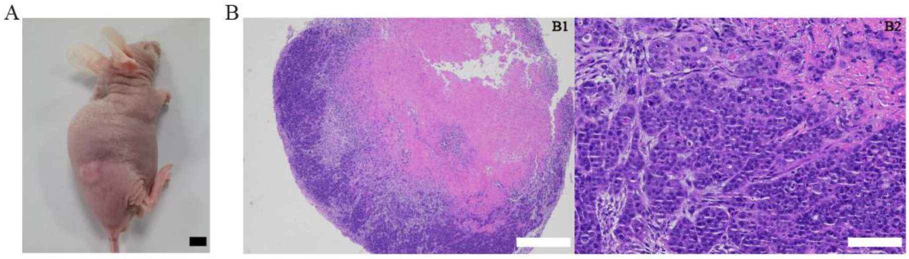

Fig. S16. The establishment of Ewing sarcoma (ES) xenograft model. (A) The appearance of ES xenograft mice model (n = 3). The tumor size reaches about 0.5×0.5 cm. Scale bar=4 mm. (B) Pathological examination carried out by H&E staining of the neoplasm shows abnormal tumor cells, large central necrosis and marginal fibrosis (n = 3). Scale bar =1 mm (40×, B1). Scale bar = 200 μm (100×, B2).

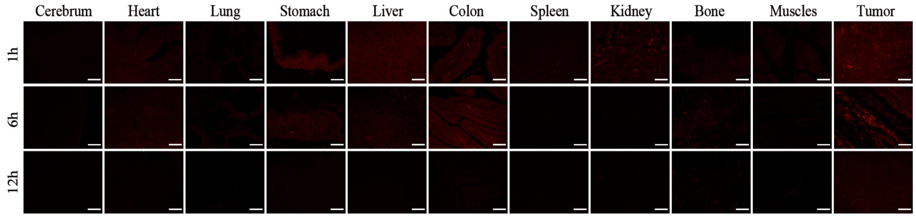

Fig. S17. Fluorescence imaging of tumor and normal tissues slices at different time points. N = 3. Scale bar = 100 μm. λex = 633 nm, slit: 10/10 nm.

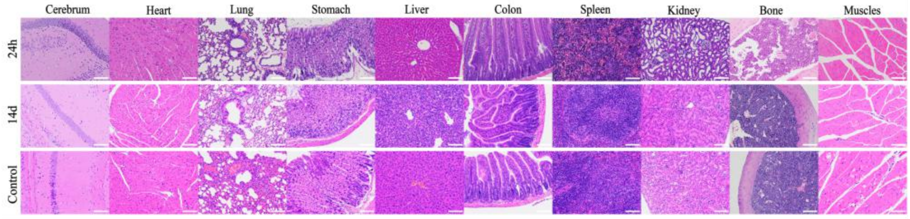

Fig. S18. The biocapacity assays was carried out by tissue slice histopathology analysis. N = 3. Scale bar = 200 μm.

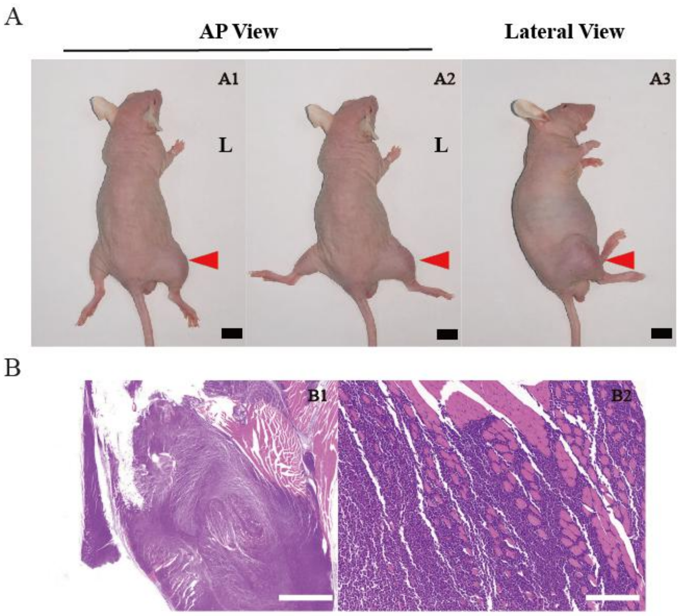

Fig. S19. The establishment of orthotopic Ewing sarcoma (ES) model. (A) Representative gross images of left knee joint lesions on the mice. Both AP view and lateral view showing a swollen kneen joint, A2 showing restriction of left knee joint activity (n = 3). Scale bar = 4 mm. (B) Orthotopic tumor biopsy shown the overall tissue was disrupted by ES tumors, and the tumor invasion to surrounding muscle tissue (n = 3). Scale bar = 2 mm (10×, B1). Scale bar=200 μm (100×, B2).

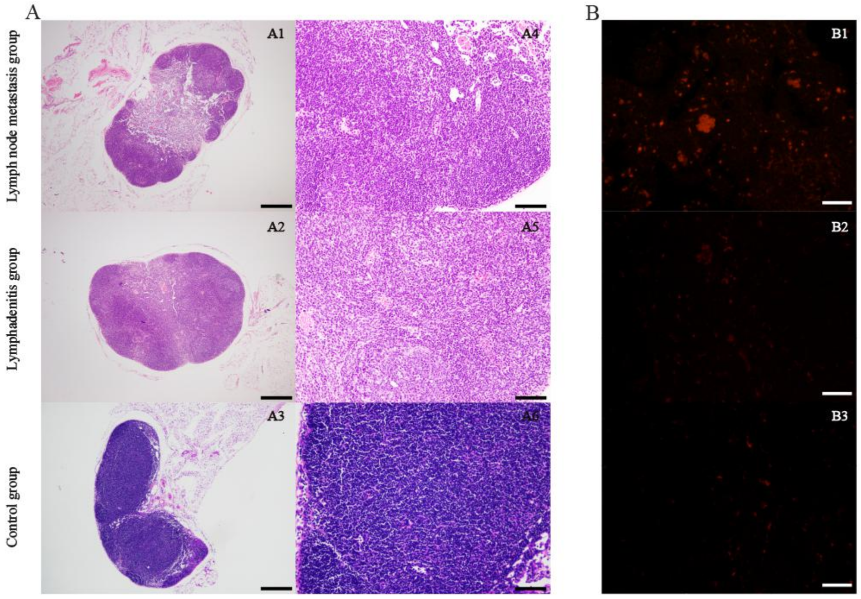

Fig. S20. The establishment of Ewing sarcoma (ES) lymph node metastasis (LNM) and lymphadenitis in MRL/MpJ mice. (A) Representative histopathology analysis performed on lymph node metastasis tissue (top row), lymphadenitis tissue (middle row) and normal lymph node tissue as a control (final row). n = 3 for each group. Scale bar = 500 μm (40×, A1, A2, A3); Scale bar = 200 μm (200×, A4, A5, A6). (B) Fluorescence imaging of inguinal lymph node tissues of different experimental groups (n = 3). Scale bar =100 μm. Λex = 633 nm, slit: 10/10 nm.
